# The Roles of Immune Cells in Gastric Cancer: Anti-Cancer or Pro-Cancer?

**DOI:** 10.3390/cancers14163922

**Published:** 2022-08-14

**Authors:** Asif Sukri, Alfizah Hanafiah, Nik Ritza Kosai

**Affiliations:** 1 Integrative Pharmacogenomics Institute (iPROMISE), Universiti Teknologi MARA (UiTM), Bandar Puncak Alam, Shah Alam 43200, Malaysia; 2 Department of Medical Microbiology and Immunology, Faculty of Medicine, Universiti Kebangsaan Malaysia, Cheras, Kuala Lumpur 56000, Malaysia; 3 Department of Surgery, Faculty of Medicine, Universiti Kebangsaan Malaysia, Cheras, Kuala Lumpur 56000, Malaysia

**Keywords:** gastric cancer, immune cells, immune response, T cells, B cells, macrophages, natural killer cells, dendritic cells

## Abstract

**Simple Summary:**

Gastric cancer is still one of the leading causes of death caused by cancer in developing countries. The emerging role of immunotherapy in cancer treatment has led to more research to elucidate the roles of essential immune cells in gastric cancer prognosis. We reviewed the roles of immune cells including T cells, B cells, dendritic cells, macrophages and natural killer cells in gastric cancer. Although the studies conducted on the roles of immune cells in gastric cancer pathogenesis produced conflicting results, understanding the roles of immune cells in gastric cancer will help us to harness them for application in immunotherapy for better prognosis and management of gastric cancer patients.

**Abstract:**

Despite the fact that the incidence of gastric cancer has declined over the last decade, it is still the world’s leading cause of cancer-related death. The diagnosis of early gastric cancer is difficult, as symptoms of this cancer only manifest at a late stage of cancer progression. Thus, the prognosis of gastric cancer is poor, and the current treatment for improving patients’ outcomes involves the application of surgery and chemotherapy. Immunotherapy is one of the most recent therapies for gastric cancer, whereby the immune system of the host is programmed to combat cancer cells, and the therapy differs based upon the patient’s immune system. However, an understanding of the role of immune cells, namely the cell-mediated immune response and the humoral immune response, is pertinent for applications of immunotherapy. The roles of immune cells in the prognosis of gastric cancer have yielded conflicting results. This review discusses the roles of immune cells in gastric cancer pathogenesis, specifically, T cells, B cells, macrophages, natural killer cells, and dendritic cells, as well as the evidence presented thus far. Understanding how cancer cells interact with immune cells is of paramount importance in designing treatment options for gastric cancer immunotherapy.

## 1. Introduction

Gastric cancer remains the fifth most common cancer worldwide, with over a million new cases having being diagnosed in 2020 alone, and it is the third leading cause of cancer-related deaths worldwide, accounting for approximately 768,000 deaths in 2020 [1]. Gastric cancer is associated with a number of factors, including the host’s genetics, *Helicobacter pylori* infection, diet, socioeconomic status, and lifestyle [2]. Gastric cancer has a poor prognosis, with the majority of patients being diagnosed at an advanced stage of the disease, particularly in developing countries [3]. The treatment of gastric cancer includes surgery for early gastric cancer and chemotherapy for advanced gastric cancer [4]. The immune response of the host is critical for the immune surveillance of cancer and immunoediting [5]. The importance of the immune system in cancer surveillance has been shown in animal models, where mice deficient in the interleukin-12β2 receptor, which is an important receptor in the interferon-γ pathway, develop lung cancer [6]. In a human study, some patients with lymphoma had a mutation in the perforin gene [7]. Cancer immunoediting is the interaction between immune cells and tumor cells for the protection, formation, or enhancement of the cancer cells. Cancer immunoediting can be divided into three phases: (1) the elimination phase, (2) the equilibrium phase, and (3) the escape phase [8]. In the elimination process, the immune system uses innate and adapted immunity to recognize and kill cancer cells, with the elimination mechanism involving cytokines such as interferon γ [9] and immune cells such as natural killer cells, T cells, and B cells [10]. Studies have shown that the equilibrium stage between cancer cells and immune cells can either suppress or enhance tumor development [11]. Furthermore, the escape process includes the loss of major histocompatibility complex (MHC) class I from cancer cells, which decreases the capacity of cytotoxic cells to detect cancer cells, as well as a rise in oncogene mutations and the development of a tumor microenvironment [12]. Immunotherapy is the manipulation of the immune system as a cancer treatment, and it presents as one of the most attractive new strategies for the treatment of gastric cancer. Examples of immunotherapy include cancer vaccines for the activation and development of T cells, monoclonal antibodies, and RNA-based vaccines [13]. In gastric cancer, the monoclonal antibody trastuzumab, which targets HER2, has been employed together with chemotherapy agents, with promising outcomes of good prognosis in the patients [14]. Moreover, the use of cytotoxic cells that express CD3^+^CD56^+^ and that are stimulated with cytokines show promising outcomes in patients with gastric cancer [15]. Thus, an understanding of how immune cells are stimulated for the treatment of gastric cancer is pertinent to designing strategies for the manipulation of cells as future medical strategies for gastric oncology. However, the roles of immune cells in gastric cancer pathogenesis remain to be elucidated. This review’s objective was to review the functions of immune cells, namely T cells, B cells, natural killer (NK) cells, macrophages, and dendritic cells in the pathogenesis of gastric cancer.

## 2. Prevalence and Incidence of Gastric Cancer Worldwide

Although the worldwide trend of gastric cancer has been observed to decline and level off in most countries in the recent decade, it is still one of the leading causes of cancer-related mortality in the world. It is estimated that the worldwide burden of gastric cancer affects 22.2 million people in 2019 [16]. The top five regions with high incidence of gastric cancer include Eastern Asia (Japan, Mongolia, Republic of Korea, and China), Eastern Europe, South America, Western Asia, and Southern Europe [1]. Over decades, the incidence and age-standardized mortality of gastric cancer has been shown to decline in developed countries, namely the USA, France, Germany, the UK, Sweden, and Switzerland [17]. Mortality and incidence of gastric cancer were high in the Republic of Korea and Japan, but continuous effort for early screening and eradication of *H. pylori* infection has contributed to the decline of the cancer in these countries in recent years [18]. Nevertheless, the rise of antibiotic resistance of *H. pylori* to essential antibiotics for treatment purposes that has been observed in recent years has complicated the prevention strategy of gastric cancer, particularly in developing countries [19]. In China, the prevalence of gastric cancer is showing an overall downward trend among patients aged more than 50 years old. However, it is important to note that an upward trend of gastric cancer incidence among patients aged between 15 to 49 years old (male and female) in China has been observed in that country from 1990 to 2019 [20], and China makes up 48.6% of the current global mortality rate of gastric cancer [21]. The incidence and mortality of gastric cancer are still high in South American countries [1] and in Mongolia, where the mortality rate of gastric cancer in that country is the highest in the world [22]. Different aetiology of gastric cancer subsites, namely cardia and non-cardia gastric cancer, has been noted. Incidence rates of cardia and non-cardia gastric cancer have been demonstrated to be different according to geographical regions in recent years. In 2012, the incidence of non-cardia gastric cancer was ~2.6 times higher than cardia gastric cancer, with the highest incidence of both cardia and non-cardia gastric cancer observed in Eastern and Southeastern Asian countries. A higher incidence of cardia gastric cancer was noted in men than women, and the incidence of cardia gastric cancer was observed to increase in certain countries such as the USA and The Netherlands [23]. In 2018, a changing trend of non-cardia vs. cardia gastric cancer was observed in which the incidence of non-cardia gastric cancer diagnosis was 4.7 times higher than cardia gastric cancer (853,000 vs. 181,000). The incidence of non-cardia and cardia gastric cancer was still observed to be the highest in Eastern Asian countries, while the incidence rates of non-cardia and cardia gastric cancer were observed to be the lowest in North American countries and Sub-Saharan African countries, respectively [24].

## 3. Pathogenesis of Gastric Cancer

Factors such as *H. pylori*, a high salt intake, a lack of ascorbic acid, and carotenoids accelerate the transformation of normal tissues toward atrophic gastritis and intestinal metaplasia [25]. Gastric cancer pathogenesis at the tissue level begins with the transformation of normal tissue to non-atrophic gastritis, multifocal atrophic gastritis without intestinal metaplasia, intestinal metaplasia with complete intestinal type, intestinal metaplasia without complete colonic type, low-grade dysplasia, high-grade dysplasia, and eventually adenocarcinoma [26]. Another factor that causes gastric cancer is chromosomal instability, which is caused by the loss or addition of chromosomes due to the perturbance of chromosomal microtubule attachment to kinetochores during mitosis [27], or changes to part of the chromosome caused by a loss of heterozygosity, gene deletion, and gene amplification [28]. A whole-genome analysis of gastric cancer tissue revealed that 50% of gastric cancer tissue harbors chromosome instability, in which tumors isolated from the cardia site demonstrated the highest frequency of chromosome instability. In contrast, intestinal-type gastric cancer demonstrates the lowest frequency of chromosome instability. Tumors with chromosomal instability also show a high mutation rate for TP53, tyrosine kinase receptor gene amplification, and high levels of epidermal growth factor receptor phosphorylation [29]. The most frequently amplified chromosome in gastric cancer is chromosome 7 (20%) [30]. Chromosome instability can also predict gastric cancer prognosis based on lymph node metastasis [31], despite the fact that another study found no connection between chromosome instability and gastric cancer prognosis [29].

Microsatellite instability in gastric tumors with hypermutation characteristics, a deficiency in gene amplification, and a frequent occurrence in older adults and women have also been observed [29]. However, this molecular type of gastric tumor was not correlated with gastric cancer prognosis [29,32], indicating that molecular instability occurs during the early pathogenesis of gastric cancer [32]. Patients with intestinal tumors, distal-type tumors, a differentiated tumor type, and a low-grade tumor were observed to harbor tumors with high levels of microsatellite instability [33]. A meta-analysis study found a correlation between tumor microsatellite instability and improved gastric cancer prognosis, in which patients with microsatellite instability demonstrated reductions in lymph node metastasis and tumor invasiveness [34].

Epigenetic changes, including DNA methylation and histone deacetylation, play a pivotal role in gastric cancer pathogenesis [35]. CpG methylation causes gene silencing that is associated with cell malignancy [36] and cancer [37]. In gastric cancer, infections with *H. pylori* and EBV have been associated with CpG methylation [38]. Methylation of the CpG region is also significantly associated with genes that are involved in cancer pathways, the Hedgehog pathway, and cytokine receptor interaction [39]. A comparison of gastric mucosa samples collected from healthy controls and subjects infected with *H. pylori* revealed a higher degree of methylation at CpG islands in subjects infected with *H. pylori* compared to healthy controls. This study also found a higher degree of methylation at CpG islands in patients with gastric cancer than in healthy controls [40]. Histone acetylation and deacetylation are essential regulators in gene transcription and silencing, in which the deregulation of this molecular mechanism contributes to cancer pathogenesis [41]. The gene expression of histone deacetylase 1 was observed to be higher in gastric tumors than in normal tissue, while the expression of the histone acetylase gene was observed to be lower in normal tissues than in gastric tumors [42]. The histone deacetylase gene is an antagonist towards TP53, an essential regulator in the cell cycle [43]. Histone deacetylase 2 gene expression, a vital gene in gastric cancer metastasis, was higher in gastric tumors than in normal tissues [44]. *H. pylori* have been shown to regulate gene expression through the acetylation process of histone remodeling. Decreased levels of histone H3 lysine 23 acetylation were observed in gastric cancer cells infected with *cag*PAI-positive *H. pylori* [45]. Phosphorylation or dephosphorylation of histone is also essential in gene expression. Dephosphorylation of histone H3, which is associated with gene repression, was observed in gastric cancer cells infected with *H. pylori* harboring *cag*PAI genes [45]. In addition, another study found that the phosphorylation level of histone H3 decreased after infection with *H. pylori* and that mechanism depends on the type IV secretion system (T4SS) expressed by genes on *H. pylori cag*PAI [46].

The risk of gastric cancer is also associated with the polymorphism of genes encoding cytokines, although some studies have found an association between cytokine gene polymorphism and gastric cancer, while others did not find an association. Interleukin-1 beta (IL-1) [47], IL-6 [48], IL-8 [49], IL-17 [50], and tumor necrosis factor (TNF-α) [51] polymorphisms have been linked to an increased risk of gastric cancer in certain populations.

## 4. Functions of Immune Cells in Gastric Cancer

### 4.1. T Cells

T cells have different categories: cytotoxic T cells (CD8+ cells), T helper cells (CD4+ cells), and regulatory T cells. However, there are T cell categories that are yet to be characterized or discovered for their functions. Several types of T helper cells, based on their cytokine secretion, include T helper cell 1 (Th1), T helper cell 2 (Th2), T helper cell 9 (Th9), T helper cell 17 (Th17), T helper cell 22 (Th22), and T helper cells with high levels of expression for forkhead box 3 (FOXP3) and CD25 [52]. For instance, Th1 produces interferon-gamma (IFN-γ) and interleukin-2 (IL-2), while Th2 produces interleukin-4 (IL-4), interleukin-5 (IL-5), interleukin-6 (IL-6), and interleukin-10 (IL-10). Those cytokines play an essential role in activating cell-mediated immunity and orchestrating B-cell mediated humoral immunity [53]. Th9 cells secrete interleukin-9 (IL-9), which involves the orchestration of cell-mediated immunity [54], while Th17 cells produce interleukin-17 (IL-17), interleukin-21 (IL-21), and interleukin-22 (IL-22), which are pertinent in combating extracellular pathogens [55]. On the other hand, Th22 secretes interleukin-22 (IL-22), which is an essential activator of innate immunity in epithelial cells [56]. Generally, the functions of T cytotoxic cells include the neutralization of host cells that are infected with intracellular pathogens, such as viruses and some bacteria, and killing cancer cells. In contrast, the function of T helper cells primarily involves the crucial neutralization of cells that have been infected with intracellular pathogens through the mediation of B cells to produce antibodies, the activation of macrophages for bactericidal activity, and the facilitation of other immune cells’ activities in orchestrating immune defense.

**Figure 1:**
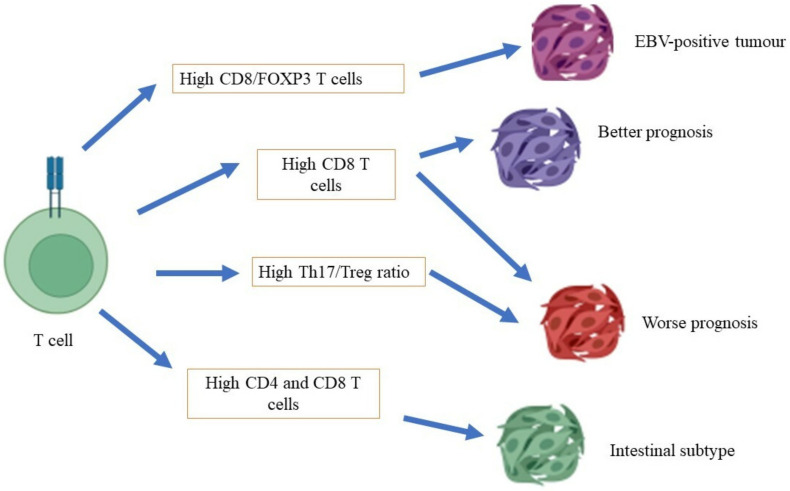
Role of T cells in gastric cancer.

Conflicting reports have been made with regard to the role of T cytotoxic cells in the carcinogenesis of gastric cancer (Figure 1). At one end of the spectrum, the distinct expression of T cytotoxic cells has been associated with a different prognosis of gastric cancer, in which a high density of T cells in patients with gastric cancer is associated with a poor prognosis of gastric cancer. A study conducted by Thompson et al. [57] showed that patients with a poor prognosis demonstrated a higher infiltration of CD8+ T cells in gastric tumors compared to patients with a better prognosis. They also found that gastric cancer cells that had been isolated from patients with a poor prognosis also expressed high levels of programmed cell death ligand 1 (PDL-1), which is a critical ligand that deactivates T cells via the stimulation of T regulatory cells, and subsequently encourages autoimmunity and the incidence of autoimmune diseases [58]. Of note, a high expression of CD8+ T cells has been shown in mixtures of cells that have been isolated from the tumors of patients with gastric cancer who were infected with the Epstein–Barr virus (EBV) [59]. At the other end of the spectrum, a study conducted by Lee et al. [60] showed that a high level of infiltration by CD8+ T cells in patients with gastric cancer was associated with a better rate of survival than for patients with a low degree of infiltration by CD8+ T cells. In the mouse model, a high infiltration of CD8+ T cells, with the absence of CD4+ T cells, has been associated with severe gastritis. This finding concludes that CD4+ T cells are vital for the surveillance of CD8+ T cell activity [61].

The balance of Th1/Th2 is paramount for *H. pylori* infection, and polymorphonuclear cells of the gastric mucosa from mice infected with *H. pylori* have been found to secrete a high level of IFN-γ and IL-4. Additionally, mice deficient in IL-4 demonstrate a phenotype of severe gastritis compared to wild-type mice, indicating the importance of cytokine secretion by Th1 and Th2 in orchestrating gastric carcinogenesis [62]. Mice lacking Th1, IL-2, and IFN-γ, but not Th2 and IL-4, were susceptible to *H. pylori* infection. However, cytokines released by Th1 cells, namely IL-12 and IFN-γ, are essential in the development of chronic gastritis [63]. In patients with gastric cancer, the gene expression of IFN-γ (the Th1 cytokine) was lower than the expression of IL-4 (the Th2 cytokine), suggesting that Th2 cells were more prominent than Th1 [64].

T regulatory (Treg) cells are CD4^+^ T cells with characteristics such as a high level of CD4, FOXP3, and CD25 expression. The primary functions of Treg cells include the prevention of autoimmune diseases and the deactivation of allergy reactions [65]. The profiling of T cells from the peripheral blood of patients with gastric cancer with different rates of prognosis revealed a high density of Treg cells in patients with a worse prognosis than those of patients with a good prognosis and of healthy subjects, in which Treg cells (CD4^+^/CD25^+^) expressed a higher level of IL-10 secretion than CD4^+^/CD25^−^ cells [66]. A different result was observed in another study, in which a high density of Treg in patients with gastric cancer was associated with a better prognosis (a low TNM stage and a low rate of tumor invasion) than in patients with gastric cancer with a low density of Treg [67]. A Treg cell subset with a high level of T-cell receptor co-stimulatory receptor (ICOS) expression was isolated from patients with late-stage gastric cancer and not from patients with early-stage gastric cancer, apart from the high rate of *H. pylori* infection in patients with the presence of this Treg subset [68]. On the other hand, an imbalance between Th17 and Treg has been associated with gastric cancer development. A high Th17/Treg ratio was found in patients with gastric cancer with high lymph node metastasis, and high levels of Th17 and Treg cells, compared to patients in the control group [69].

### 4.2. B Cells

B cells function as antigen-presenting cells, and they can differentiate into plasma cells to produce antibodies and to secrete cytokines such as IL-6, IFN-γ, and tumor necrosis factor α (TNF-α), for the development of CD4+ effector and memory T cells [70]. A subset of B regulatory cells (Breg) has been shown to inhibit antitumoral activity that is mediated by T cells and other immune cells, namely effector T cells, natural killer cells, and tumor-associated macrophages [71]. A comprehensive immunophenotype of gastric cancer cells using an antibody microarray showed a higher expression of B-cell markers, including CD19, CD38, and surface immunoglobulin (sIg), in patients with *H. pylori*-infected gastric cancer than in patients without infections [72].

### 4.3. Macrophages

**Figure 2:**
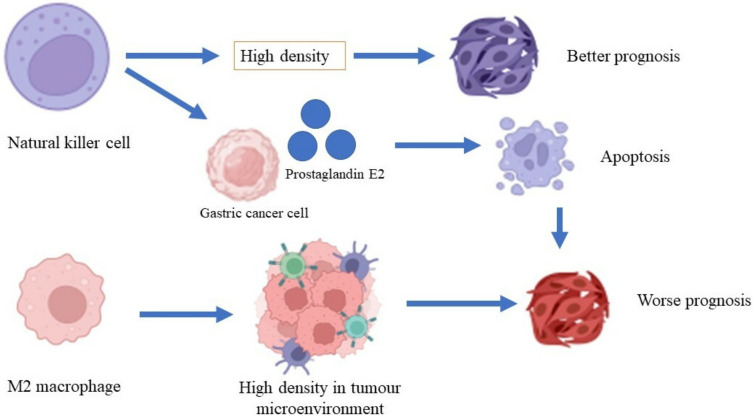
Roles of macrophages and natural killer cells in gastric cancer.

Macrophages are phagocytes that play a pivotal role in the clearance of erythrocytes and tissue modeling [73]. Furthermore, these cells also have an essential role in recognizing pathogens via receptor recognition and the neutralization of apoptotic cells [74]. A high density of macrophages is associated with a low rate of survival in patients with gastric cancer and increased gastric tumor invasiveness through the β-catenin pathway (Figure 2) [75]. A high density of tumor-associated macrophages (TAM) of phenotype M2 was found in patients with gastric cancer with invasive gastric peritoneum compared to patients without invasive gastric peritoneum. Additionally, a gastric cancer cell line co-cultured with the TAM phenotype M2 also showed increased degrees of invasiveness and cell division in the xenographic model [76]. Osteopontin, a protein that is involved in inflammation and cancer pathogenesis [77], recruits macrophages into the tumor microenvironment and polarizes macrophages to the M2 phenotype, which facilitates gastric cancer development [78]. On the other hand, macrophages induce invasiveness, migration, and gastric cancer cell movement through epidermal growth factor and the phosphorylation of Akt, Erk ½, and c-Src [79]. *H. pylori*-infected macrophages demonstrated the activation of immune-regulated genes such as Cd44, Cd40, Cd86, and Cd274, and the inhibition of macrophage cell line division, primary macrophage cells, and the transcription of genes that are involved in DNA synthesis and the cell cycle [80].

### 4.4. Natural Killer Cells

Natural killer cells are lymphocytes that are important in mediating innate immunity and adaptive immunity for the surveillance of the immune system against invading intracellular viruses and cancer cells [81]. Natural killer cells can recognize cancer cells because they express natural killer group-2 (NKG2D), which is expressed by cancer cells [82]. The expressions of CD antigens such as CD56 and CD16 play essential roles in mediating the activation of immune cells, including macrophages, T cells, and dendritic cells [83]. A study conducted by Ishigami et al. [84] revealed that a high density of natural killer cells in patients with gastric cancer is associated with a better prognosis of gastric cancer compared to a low density of natural killer cells. A lysis activity that is greater than 25% in natural killer cells is significantly associated with a better five-year survival rate compared to lysis activity that is less than 25% [85]. A high apoptosis rate for natural killer cells was shown in patients with advanced-stage gastric cancer. However, the rate of apoptosis was reduced after the patients underwent gastrectomy [86]. Gastric cancer cells produce prostaglandin E2, which inhibits natural killer cell division and encourages apoptosis [87].

### 4.5. Dendritic Cells

Dendritic cells, or antigen-presenting cells, are master regulators of innate immunity and adaptive immunity because they can present antigens to major histocompatibility complex (MHC) class I and MHC class II. Immature dendritic cells express low levels of CD54, CD58, CD80, CD86, CD40, CD25, CD83, and IL-12, whereas mature dendritic cells express those CD antigens at high levels. IL-12 is an essential cytokine for activating natural killer cells, T cells, and B cells [88]. There are several types of dendritic cells, including myeloid dendritic cells, plasmacytoid dendritic cells, Langerhans cells, microglia cells, and CD14+ dendritic cells, which are based on the cell markers that are expressed on their surfaces [89]. In ovarian cancer cells, dendritic cells express high levels of B7-H1 and PD-1, which inhibit T-cell activation and deactivate the function of dendritic cells in recognizing tumors [90]. Factors that are secreted by tumor cells inhibit the differentiation of dendritic cells, which deactivates their antitumoral properties [91]. Furthermore, factors that are produced by tumor cells leads to the high expression of CD11b, which reduces the expression of MHC class II and is associated with a worse prognosis in patients with gastric cancer [92].

## 5. Immune Checkpoints in Gastric Cancer

Immune checkpoints are essential to maintain the self-tolerance of the immune system to body cells and prevent autoimmune reactions. Tumour cells exploit the immune checkpoint molecules to their advantage by downregulating the surveillance of immune cells toward the cancer cells [93]. Immune checkpoint molecules are often expressed on immune cells as master regulators to keep the immune system in check. Immune checkpoint molecules that have been extensively studied include programmed cell death receptor-1 (PD-1), programmed cell death receptor ligand-1 (PD-L1), programmed cell death receptor ligand-2 (PD-L2), lymphocyte activation-gene-3 (LAG-3), cytotoxic T lymphocyte antigen-4 (CTLA-4), B and T lymphocyte attenuator (BTLA) [94]. Furthermore, an immune checkpoint score based on twenty immune checkpoints has been studied and can be applied to predict the prognosis of gastric cancer patients [95]. Cancer cells express PD-L1 to escape immune surveillance through binding to PD-1 expressed on activated immune cells, namely T cells, monocytes, macrophages, and natural killer cells. The binding inhibits paramount immune signal transduction pathways and renders immune cells to be unresponsive to cancer cells and causes the downregulation of immune cell proliferation, cytokine secretion, and activation [96].

Treatments of gastric cancer include curative resection and adjuvant chemotherapy depending on the staging of gastric cancer [97]. Immunotherapy through the identification of immune checkpoints that can be manipulated to attack cancer cells has emerged as an alternative treatment for gastric cancer. Immune checkpoint inhibitors have been introduced to activate immune cells in gastric cancer patients as part of immunotherapy. Several monoclonal antibodies, namely nivolumab and pembrolizumab, that target the PD-1/PD-L1 immune checkpoint have undergone clinical trial phases with promising results. In a phase 3 clinical trial conducted among gastric cancer patients at an advanced stage from East Asian countries (Japan, South Korea, and Taiwan), the administration of nivolumab increased median follow-up in surviving patients, median overall survival, and the 1-year overall survival rate in the nivolumab group compared to the placebo group. However, adverse events were more frequently observed in the group that received nivolumab than placebo [98]. Another monoclonal antibody that targets the PD-1/PD-L1 checkpoint is pembrolizumab. A phase 1b clinical trial conducted among advanced gastric cancer patients who were positive for PD-L1 found that pembrolizumab had manageable toxicity safety among patients and elicited antitumoral activity against gastric cancer [99]. Another drug that targets the PD-1 checkpoint is avelumab. A phase 3 clinical trial that involved advanced gastric cancer patients found that avelumab did not improve overall survival and progression-free survival of the patients compared to chemotherapy, although the toxicity of avelumab was more manageable than chemotherapy [100]. A combination of monoclonal antibodies that target PD-1 (nivolumab) and CTLA-4 (ipilimumab) have also been used in clinical trials. Recent global phase 3 clinical trials (CheckMate 649 study) that compared the combination of nivolumab and ipilimumab to that of nivolumab and chemotherapy revealed that the combination of nivolumab and chemotherapy resulted in better overall survival of the patients than that of nivolumab and ipilimumab [101].

## 6. Molecular Subtypes and Immune Cells in Gastric Cancer

Whole genome sequencing of gastric cancer has provided insights on molecular subtypes of gastric cancer that can be divided into four subtypes i.e., microsatellite unstable tumour, EBV-positive tumour, tumour with chromosomal instability, and genomically stable tumour [29]. A different molecular subtype of gastric cancer has been shown to have a different prognosis, in which EBV-positive subtype has the best prognosis while genomically stable subtype has the worst prognosis [102]. The immune response in gastric cancer patients based on molecular subtypes of the tumour has been studied. In EBV-positive tumors, the high number of T cell infiltration with a high ratio of CD8^+^/FOXP3^+^ T cells was observed, while a low level of T cell infiltration was observed in *TP53* aberrant subtype tumors [103]. Similar findings were also noted in another study whereby EBV-positive tumors harboured high T cell infiltration compared to intestinal and diffuse tumors. Furthermore, the authors also observed high the infiltration of macrophages and T cells in tumors with positive EBV and high microsatellite instability [104]. A recent study divided gastric tumors with different immune activities and genomic profiles into three different immune subtypes, namely immune-deprived tumors, immune-enriched tumors and stromal-enriched tumors. Immune-deprived tumors had the lowest immune activities of cytolytic activity, lymphocyte infiltration, and immune scores as compared to immune-enriched tumors. In addition, immune-deprived tumors displayed significantly higher *TP53* mutation than that of stromal-enriched and immune-enriched tumors [105]. Similar studies also showed that gastric cancer patients with immune-enriched tumors exhibited the best prognosis, followed by immune-deprived patients, and stromal-enriched patients [105]. A study conducted to classify gastric tumors based on 390 immune gene expressions found that gastric tumors can be divided into two types, namely C1 and C2. The C2 tumour type consisted of important activated immune cells such as NK cells, CD4 memory T cells, mast cells, and dendritic cells, while in the C1 tumour type, B cells, dendritic cells, and memory T cells were at a resting state. Not surprisingly, patients with C2 tumors had better prognosis than that of patients with C1 tumors [106]. Chen et al. [107] classified gastric tumors into three subtypes: i.e., immune subtype 1, 2 and 3, in order to determine the prognosis of gastric cancer patients based on the immune landscape of gastric tumors. They found that patients with immune subtype 3 with a high infiltration of CD8^+^ and CD4^+^-activated T cells, NK cells, and M1 macrophages had the best prognosis, while the patients with immune subtype 1 with a high infiltration B cells, dendritic cells, and CD4^+^ resting T cells had the worst prognosis of gastric cancer. Taken together, the characterization and classification of patients based on gastric tumour immune landscape have yielded encouraging results to predict the prognosis of gastric cancer patients.

## 7. Immune Cell Response in Intestinal vs. Diffuse Gastric Cancer

Lauren’s classification has divided gastric cancer into two major pathological types, namely intestinal and diffuse gastric cancer. Intestinal type gastric cancer is characterized by well-differentiated cancer cells, while the diffuse type is characterized as poorly differentiated cancer cells [108]. Both types display distinct carcinogenesis, in which the intestinal type follows Correa’s gastric cancer model pathogenesis that starts with chronic gastritis, atrophy, intestinal metaplasia, dysplasia and finally gastric adenocarcinoma [25,26], while the diffuse type is closely related to the mutation of E-cadherin [109]. Furthermore, the intestinal type is more prevalent in men and elders, those with small tumor size, and better patient prognosis than that of the diffuse type [110]. Progression from normal gastric tissue to gastric adenocarcinoma involves complex underlying molecular pathways coupled with environmental factors including *H. pylori* infection and diets [111]. In the initial stage of gastric carcinogenesis, *H. pylori* initiates inflammation in normal gastric mucosa by the stimulation of gastric cells to release IL-8 and recruit neutrophils [49]. Comparing the host’s immune response according to Lauren’s classification is pertinent to help in understanding why patients diagnosed with intestinal adenocarcinoma have a better prognosis than that of patients with diffuse adenocarcinoma. Studies conducted on the role immune cells in premalignant lesions of intestinal gastric cancer and how they contribute to gastric carcinogenesis are lacking. Multiple recent studies have been conducted to unravel differences in cell-mediated immune responses between the intestinal type of gastric cancer to that of diffuse type. Recent studies showed that a higher proportion of immune cells were detected in the diffuse type than that of the intestinal type based on single cell transcriptomics analysis [112]. In addition, the early intestinal type of gastric cancer displayed more active immune cell response as compared to premalignant lesions [113]. The increased score of stem cell-like properties has also been shown in premalignant gastric lesions in comparison to normal gastric cells [113]. A higher density of natural killer cells, CD8^+^ tumor infiltrating lymphocyte and Treg cells was found in patients diagnosed with the intestinal type than that of the diffuse type. However, a similar study revealed no difference between the intestinal and diffuse types in circulating CD4^+^ and CD8^+^ T cells isolated from the peripheral blood of gastric cancer patients [114]. In contrast, another study revealed that patients diagnosed with the diffuse type of gastric cancer had a higher density of T cells (CD4^+^ and CD8^+^) than that of patients with the intestinal type. Nevertheless, they found that patients with the intestinal type had a higher density of macrophages than that of the diffuse type [104]. Inflammatory cancer fibroblasts, which have been associated with cancer cell invasiveness and stemness, were more frequently found in the diffuse type than that of the intestinal type [112].

## 8. Challenges to Associate the Immune System with Gastric Cancer

Gastric cancer is a heterogenic disease with multifactorial factors that include the environment, genetics, and the host’s immune response. The continuous discovery of novel biomarkers with pertinent roles in cancer immune response for the prognosis and diagnosis of gastric cancer is essential for further understanding of how immune response works in gastric cancer pathogenesis. With the advent of personalized medicine, treatment can be designed according to immune response and genome. The Cancer Genome Atlas has characterized gastric cancer into four subtypes, namely microsatellite unstable tumour, EBV-positive tumour, tumour with chromosomal instability, and genomically stable tumour based on whole genome sequencing [29]. This characterization has led to the promising application of personalized medicine among gastric cancer patients. Patients can be screened based on their genome and the host’s immune response to tailor the best treatment options for them. Furthermore, the application of pharmacogenomics can be applied to reduce adverse events associated with treatment administered to patients. As the incidence of gastric cancer is more prevalent in low-and-middle-income countries compared to high income countries, it is challenging to implement personalized medicine in the region with low resources, although the implementation of pharmacogenomics has been demonstrated to be economically cost-effective in developing countries [115]. Thus, the prevention of gastric cancer through the early screening and eradication of *H. pylori* currently remains pertinent in low-and-middle income regions.

## 9. Conclusions

In conclusion, there have been contradictory reports regarding the role of immune cells in the pathogenesis of gastric cancer. Immune cells have been shown to play a major role in improving the prognosis of patients with gastric cancer on one end of the spectrum, while on the other end of the spectrum the same cells have been shown to worsen patient prognosis. Although several studies have been performed on the function of T cells in gastric cancer pathogenesis, studies on B cells, dendritic cells, natural killer cells, and macrophages have been lacking. Future research should concentrate on how different types of immune cells interact within the pathogenesis of gastric cancer. Discoveries with regard to the functions of immune cells in gastric cancer could lead to the development of a therapeutic alternative that manipulates the immune system to fight cancer cells based on an individual’s immune response. This could be a potential treatment option for precision medicine and health in the treatment of gastric cancer.
